# Folding and self-association of atTic20 in lipid membranes: implications for understanding protein transport across the inner envelope membrane of chloroplasts

**DOI:** 10.1186/s12858-014-0029-y

**Published:** 2014-12-31

**Authors:** James H Campbell, Tuan Hoang, Masoud Jelokhani-Niaraki, Matthew D Smith

**Affiliations:** Department of Biology, Wilfrid Laurier University, 75 University Avenue West, Waterloo, ON N2L 3C5 Canada; Department of Chemistry & Biochemistry, Wilfrid Laurier University, Waterloo, ON Canada; Biophysics Interdepartmental Group, University of Guelph, Guelph, ON Canada; Current address: Department of Biology, University of Waterloo, Waterloo, ON Canada

**Keywords:** Tic20, TIC complex, Protein self-assembly, Circular dichroism, Protein folding, Structure-function relationship, Protein reconstitution, Chloroplast membrane proteins

## Abstract

**Background:**

The *Arabidopsis thaliana* protein atTic20 is a key component of the protein import machinery at the inner envelope membrane of chloroplasts. As a component of the TIC complex, it is believed to form a preprotein-conducting channel across the inner membrane.

**Results:**

We report a method for producing large amounts of recombinant atTic20 using a codon-optimized strain of *E. coli* coupled with an autoinduction method of protein expression. This method resulted in the recombinant protein being directed to the bacterial membrane without the addition of a bacterial targeting sequence. Using biochemical and biophysical approaches, we were able to demonstrate that atTic20 homo-oligomerizes *in vitro* when solubilized in detergents or reconstituted into liposomes. Furthermore, we present evidence that the extramembranous N-terminus of the mature protein displays characteristics that are consistent with it being an intrinsically disordered protein domain.

**Conclusion:**

Our work strengthens the hypothesis that atTic20 functions similarly to other small α-helical integral membrane proteins, such as Tim23, that are involved in protein transport across membranes.

**Electronic supplementary material:**

The online version of this article (doi:10.1186/s12858-014-0029-y) contains supplementary material, which is available to authorized users.

## Background

Tic20 is an integral membrane protein and a core component of the translocon at the inner envelope membrane of chloroplasts (TIC complex) that is involved in the import of nucleus-encoded proteins into the organelles [1,2]. Other proteins reported to be involved in protein translocation across the inner membrane include Tic110, Tic40, Tic22 and Tic21 [3,4], and the more recently identified Tic214, Tic100 and Tic 56 [5]. Tic62, Tic55 and Tic32 have been reported to play regulatory roles in protein import across the inner membrane [2]. Tic20 was originally identified in *Pisum sativum* (pea; psTic20) using chemical cross-linking, which showed that it associates with preproteins transiting the inner membrane [1,6,7]. There are four Tic20 isoforms in *Arabidopsis thaliana* (denoted atTic20-I, -II, -IV, and –V) [8-10]. atTic20-I is the dominant Arabidopsis isoform (hereafter referred to as atTic20), the most similar to the originally characterized isoform from pea [8,10,11], and is the focus of the current study. Based on the *in vitro* cross-linking data, and more recent *in vivo* evidence and bioinformatics analysis, Tic20 has been hypothesized to serve as a preprotein conducting channel of the Tic complex. In *Arabidopsis,* knock-down of atTic20 using anti-sense technology resulted in plants with a pale phenotype; the plastids of these plants were arrested at a pre-chloroplastic developmental state, and preprotein translocation was inhibited at the inner membrane [12]. More recently, atTic20-I knockout mutants (*tic20-I*) have been characterized with pale phenotypes that are consistent with the phenotype of the antisense plants [10,11,13]. atTic20 is also reported to be a core component of a 1 MDa complex isolated from the inner envelope membrane of Arabidopsis chloroplasts that is thought to comprise the channel-forming TIC complex [5,14], and electrophysiological experiments have shown that Tic20 does have some capacity to function as an ion-permeable pore in reconstituted membranes [15]. Further evidence in support of Tic20 having a role in membrane translocation comes from bioinformatic analyses, which show that it shares homology with cyanobacterial amino acyl transporters, as well as with the mitochondrial channel protein, Tim23 [9,16]. The similarity with Tim23 extends to the predicted topology of the proteins. Originally, it was proposed that psTic20 contained 3 transmembrane α-helical domains [7], but it now seems likely that it has 4 such domains [9,10,12,15], as does Tim23 [17].

Despite the evidence in support of a function for Tic20 in protein conductance across the inner membrane, direct evidence for the mechanism by which Tic20 might transport preproteins is lacking. The preprotein channel of the TOC complex, Toc75, is a β-barrel through which preproteins transit [18]. Tic20 is unlikely to transport preproteins in the same manner at Toc75, owing to its predicted α-helical conformation. That Tic20 is present in the 1 MDa complex of the inner membrane together with the newly discovered TIC components Tic100, Tic56 and Tic214 [5,14], but in the absence of Tic110, which is another TIC component that has been suggested to serve a channel function [19,20], is additional evidence in support of a role for Tic20 as a preprotein channel. The role of the new Tic components is unknown, but the study provides evidence that atTic20 provides the preprotein conduit across the inner membrane [5,14]. There is also evidence that Tic21, a protein related to Tic20, may serve a channel function, perhaps at specific stages of development [9,11,13,14]. If Tic20 does provide the pre-protein channel across the inner membrane, it may function in a manner similar to the related mitochondrial protein Tim23.

One of the structural features of Tim23 is an intrinsically disordered domain on its N-terminus that resides in the mitochondrial intermembrane space, and that has a role in precursor protein recognition [17]. Intrinsically disordered proteins with inducible structure are known to participate in protein-protein interactions [21]. The members of the Toc159 family of proteins contain large N-terminal domains (the A-domains) that have been demonstrated to be intrinsically disordered and are hypothesized to play a role in transit peptide recognition and binding [22,23]. It is of interest whether the N-terminal extramembranous region of atTic20 is also intrinsically disordered; if so, it may have a role in preprotein recognition, or in TIC complex assembly.

The current study makes use of recombinant versions of atTic20 produced in *E. coli* using an autoinducing expression system [24]. Recombinant full-length (mature) atTic20 and an N-terminally truncated version (atTic20ΔN20) were expressed in *E. coli* membranes at a high yield*.* These proteins were folded with a high helical content when extracted in mild detergents and when reconstituted into lipid vesicles. On the other hand, a synthetic peptide corresponding to the 20 N-terminal amino acids of atTic20 displayed features of intrinsically disordered proteins (IDPs). Evidence for direct self-association of atTic20 was also observed *in vitro* for the first time. Collectively, these data shed light on the role of atTic20 in the dynamics and physiology of the TIC complex.

## Results

### In silico analysis for atTic20

**Figure 1:**
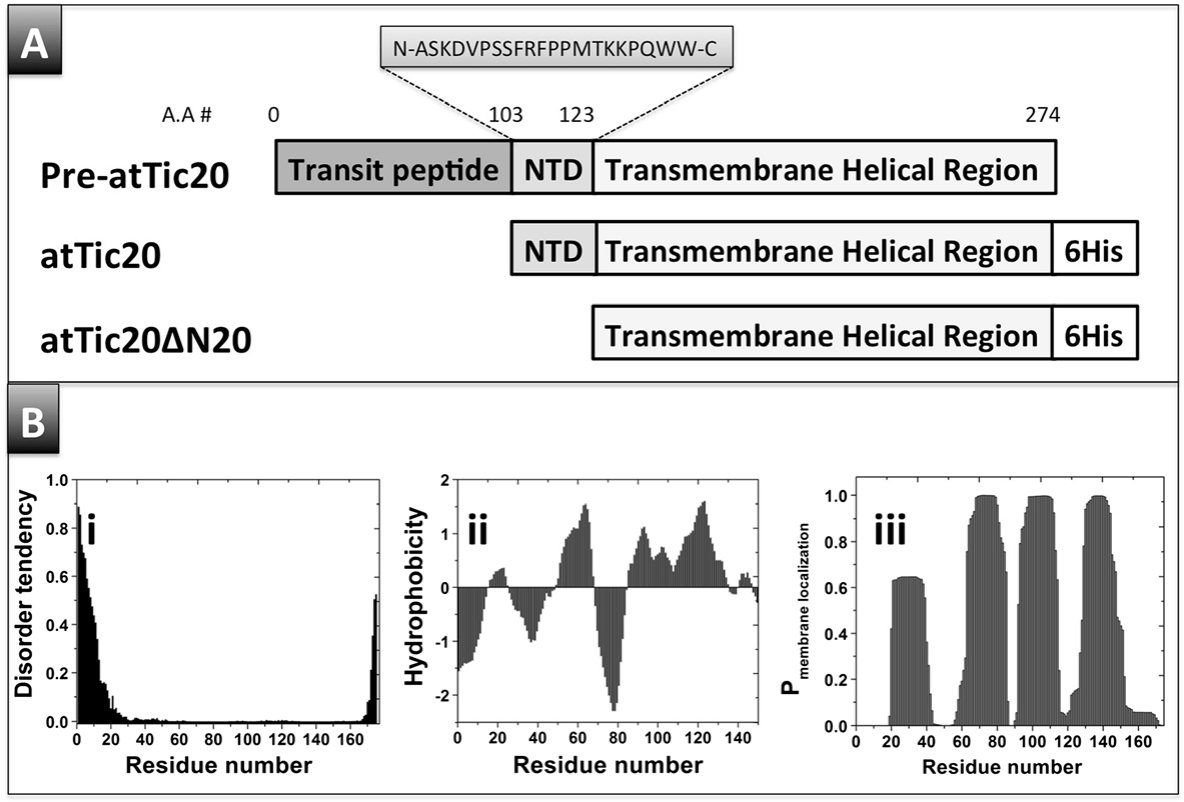
**Design of atTic20, its truncated mutant and extramembrane N-terminal peptide. (A)** Schematic representation of different forms of atTic20 used in this study. A cDNA encoding pre-atTic20 (including the transit peptide) was used to generate constructs encoding mature atTic20 and atTic20ΔN20, a truncated mutant lacking the 20 amino acid N-terminal domain (NTD), with C-terminal hexahistidine tags (6His). A 21-amino acid peptide corresponding to the NTD was also synthesized (amino acid sequence shown). **(B)** Results of *in silico* analyses suggest that atTic20 contains four transmembrane domains and that its N-terminal peptide is disordered. (i) IUPred was used for disorder predictions of atTic20. Transmembrane prediction for atTic20 was based on (ii) hydrophobicity plot and (iii) TMHMM analysis. Refer to the Methods for further detailed method and analysis.

Tic20 has been predicted to have 4 TM helices [1,10,12,15], and our *in silico* modelling using TopPred, 3D Distill servers and TMHMM are consistent with this prediction (Figure 1, Bii and Biii) [25,26]. The predictions are also consistent with atTic20 having small N- and C-terminal soluble segments [13], which we then examined for predicted intrinsic disorder using IUPred [27]. Using the short disorder parameter, IUPred predicted that the N-terminal segment has a strong tendency towards disorder (Figure 1Bi). Based on this analysis, we devised two constructs for further work: one encoding a mature, transit peptide-lacking version of atTic20 and an N-terminally truncated version (atTic20ΔN20) lacking the N-terminal amino acids of the mature protein that were predicted to form the intrinsically disordered segment (Figure 1A). Additionally, we prepared a synthetic peptide corresponding to the extramembrane N-terminal domain that was predicted to be disordered.

### Optimization of atTic20 and atTic20ΔN20 expression in bacterial membranes

**Figure 2:**
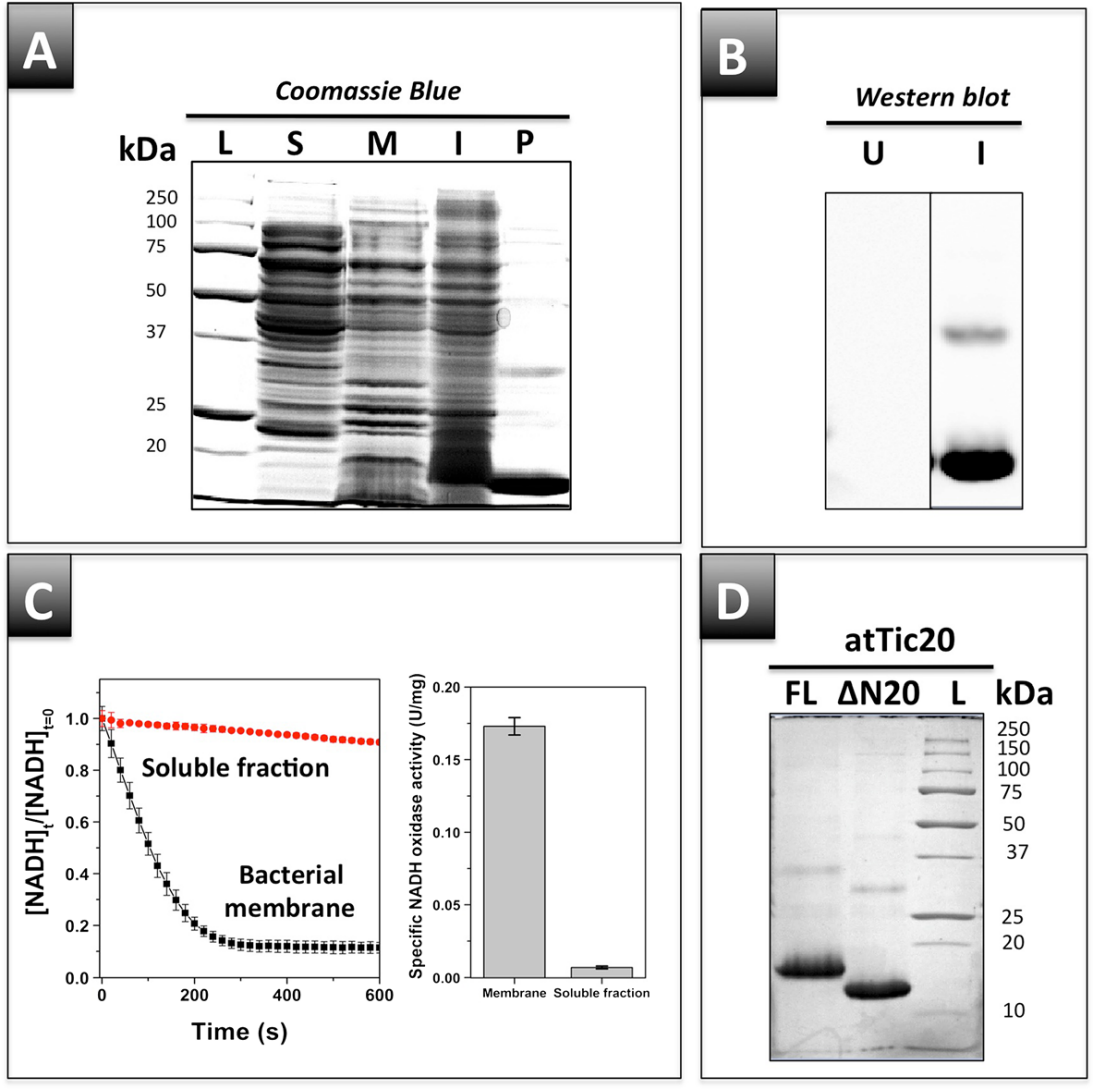
**Expression of atTic20 and its truncated mutant (atTic20ΔN20) in bacterial membranes. (A)** Fractionation of *E. coli* BL21 CodonPlus (DE3)-RIPL cells expressing recombinant atTic20 using the autoinduction method. Protein profiles of bacterial cell fractions (S, total soluble proteins; M, total membranes; I, inclusion bodies) and atTic20 purified from the membrane fraction (P) were compared using SDS-PAGE stained with Coomassie Blue. 5 μg of total protein was loaded in each lane. The left lane contains a protein molecular weight (kDa) ladder (L). **(B)** Western blot detection of recombinant atTic20 in the bacterial membrane fraction of auto-induced (I) and uninduced (U) bacterial cells using mouse IgG2b anti-histidine antibody, detected by chemiluminescence using a horseradish peroxidase-linked secondary antibody. **(C)** An assay for the membrane marker NADH oxidase was used to confirm the presence of membranes in the “membrane fraction” and to compare to the soluble protein fraction from Figure 2A. The NADH oxidase assay measures the conversion of NADH from its reduced to its oxidized form, indicated by a decrease in A_340_ over time. Average specific NADH oxidase activity (U/mg protein) of the soluble and bacterial membrane fractions are shown. **(D)** Comparison of purified atTic20 (FL) and its truncated mutant (ΔN20), resolved using semi-native PAGE (2 mM SDS in the running buffer; 0 mM SDS included in the sample buffer) stained with Coomassie blue. The molecular weights (kDa) of the protein ladder (L) are indicated.

Until recently, production of recombinant versions of Tic20 in *E. coli* had not been reported in the literature [15]. Kovacs-Bogdan et al. reported producing codon-optimized psTic20 in an *E. coli* cell-free lysate system, and atTic20 in *E. coli* BL21(DE3) in inclusion bodies [15]. In order to optimize the expression of recombinant atTic20 and atTic20ΔN20 in the current study, a variety of expression conditions were compared. Production of recombinant atTic20 in *E. coli* BL21 CodonPlus (DE3)-RIPL using IPTG under various temperatures did not produce significant yields of protein, except in inclusion bodies (data not shown). To overcome this problem, auto-induction was used as a milder expression method [24]. Expression of the recombinant versions of atTic20 in bacterial cells using the auto-induction method at RT resulted in enriched growth of the cultures. Auto-induction resulted in improved production of both recombinant atTic20 and atTic20ΔN20. Both proteins were abundant in the inclusion body fraction, as revealed on Coomassie-stained gels (Figure 2A; atTic20ΔN20 not shown). While the majority of the recombinant protein was directed to inclusion bodies (Figure 2A, Coomassie gel, lane I), it could also be detected in the bacterial membrane fraction (Figure 2A, Coomassie gel, lane M), albeit not as abundantly. The protein could be extracted from the membrane fraction using mild detergent (ZW 3-14), and purified to near homogeneity, and in good yield (approximately 1 mg/L of culture, and see Additional file 1), using Ni-affinity chromatography (Figure 2A, lane P). The identity of the recombinant protein in the membrane faction was confirmed using a Western Blot probed with anti-histidine antibody (Figure 2B). The membrane content of the fraction from which atTic20 and atTic20ΔN20 were extracted was confirmed using the bacterial membrane marker NADH oxidase (Figure 2C). Collectively, these data show that the auto-induction method resulted in production of recombinant atTic20 and atTic20ΔN20 in the bacterial membrane fraction, and that pure protein could be obtained in high yield using mild extraction and purification conditions. The two purified recombinant proteins (full length atTic20 atTic20ΔN20) are compared using semi-native PAGE stained with Coomassie blue in Figure 2D.

### Refolding of atTic20 and atTic20ΔN20 in detergents and liposomes

**Figure 3:**
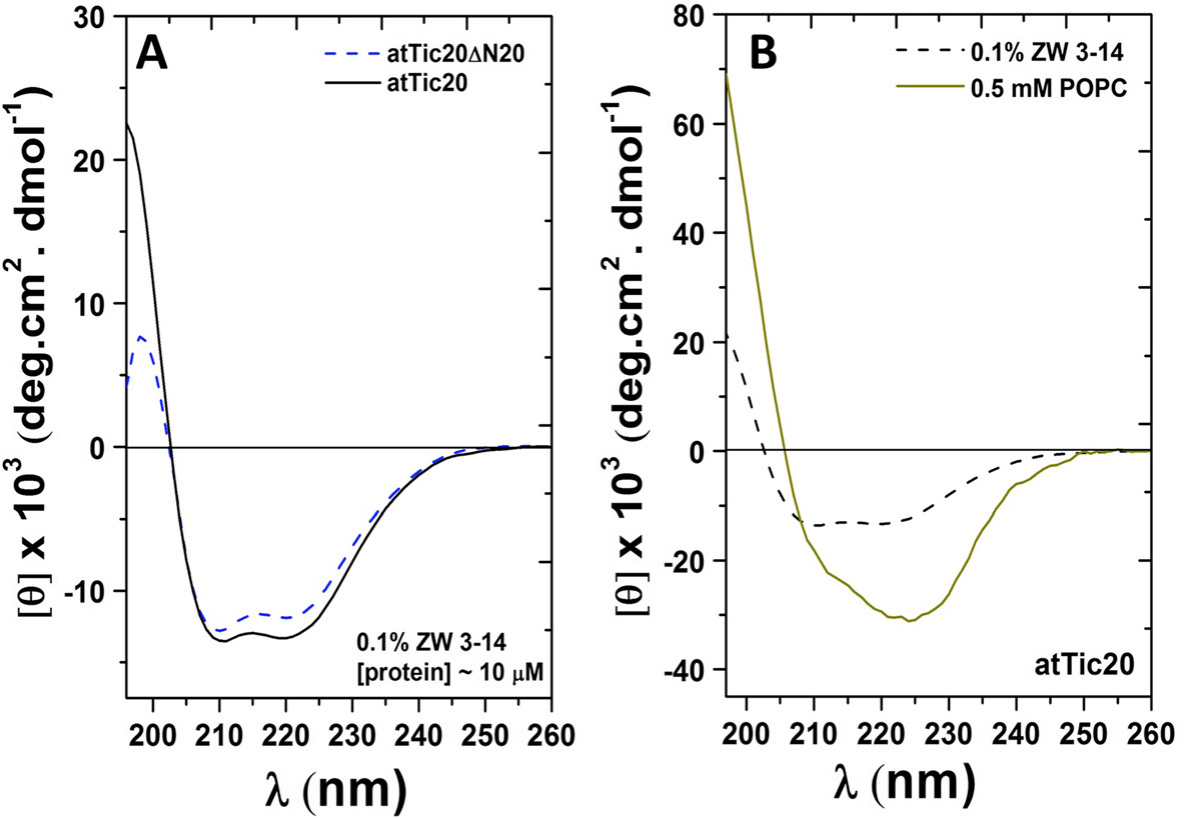
**CD analysis of atTic20 and atTic20ΔN20 in detergents and liposomes. (A)** Far-UV CD spectra of purified atTic20 and atTic20ΔN20 in 0.1% ZW 3-14 detergent. The protein concentration was ~10 μM, as determined by Bradford protein assay. **(B)** Far-UV CD of atTic20 in detergent and POPC lipid vesicles. Protein and lipid concentrations were ~ 500 nM and 0.5 mM, respectively. All samples were measured in buffer containing 20 mM Tris-HCl, 100 mM NaCl, 1% glycerol, and pH 8.0. Summary of deconvoluted data are shown in Table 1.

The secondary structure of purified atTic20 extracted from bacterial membranes using four different detergents (TX-100, LDAO, CHAPS, and ZW 3-14) at concentrations above their CMC value was compared. The secondary structure of purified atTic20 in each detergent was examined by far-UV CD spectroscopy, and the helical content for each was estimated (Table 1). Among the four detergents tested, LDAO and TX-100 induced the least helical content in atTic20 (estimated to be less than 20%; Table 1). In addition, the protein exhibited a low stability in these detergents, resulting in protein aggregation/precipitation at 4°C (data not shown). The far-UV CD spectra of atTic20 in 1% CHAPS indicated the protein had 40% α-helical content, with local minimum ellipticities at 208 nm and 222 nm. The protein, however, also displayed a low level of stability in this detergent. The protein extracted and purified in 0.1% ZW 3-14 exhibited the highest α-helical content of the detergents tested, with local minimum ellipticities at 222 nm and 208 nm, and a positive maximum ellipticity near 195 nm (Figure 3A). Deconvolution of the CD spectrum of the protein in 0.1% ZW3-14 revealed a 47% α-helical content (Table 1). In addition, atTic20 was solubilized from the bacterial membrane fraction much more efficiently with ZW 3-14, as compared to the other detergents, which resulted in this detergent providing the highest yield (~1 mg purified protein/L of culture). Collectively, the data suggested that atTic20 remained folded in a native-like functional form when extracted from bacterial membranes using 0.1% of the ZW 3-14 detergent. This condition was also used successfully to isolate and purify atTic20ΔN20 (Figures 2D and 3A). Although lacking the 20-amino acid N-terminal domain, purified atTic20ΔN20 in 0.1% ZW 3-14 still exhibited a highly helical secondary structure, similar to the full-length version (Figure 3A). The far-UV CD spectra of atTic20ΔN20 also displayed minima at both 222 nm and 208 nm, which are characteristics of helical proteins. Deconvolution of the CD spectra of atTic20ΔN20 revealed a 43% helical content of the protein in 0.1% ZW 3-14 (Table 1). Overall, our detergent screening revealed ZW 3-14 to be a suitable detergent for extracting and purifying atTic20 and atTic20ΔN20, providing a stable protein-detergent mixed micelle environment for structural and functional studies.

**Table 1:** **Secondary structure composition of atTic20 and its truncated mutant in detergents and lipid vesicles** ^**a**^

**Proteins**	**Environment**	**α**	**β**	**Turn**	**Random**	**NRMSD**
atTic20	CHAPS micelles	40	11	14	27	0.036
	LDAO micelles	7	30	19	43	0.035
	TX- 100 micelles	19	25	20	36	0.032
	ZW 3-14 micelles	47	12	18	23	0.028
	POPC vesicles	74	11	5	9	0.002
atTic20	Distill prediction*	69	--	--	--	------
atTic20ΔN20	ZW 3-14 micelles	43	20	16	21	0.013

^a^Deconvolution of CD spectra was performed using the CDSSTR program on the Dichroweb website (See Methods). The values represent the percentage of secondary structure composition. NRMSD, normalized root mean square deviation, denotes the best fit between the calculated and experimental CD spectra.

*Helical content of atTic20 was predicted using the Distill prediction program, as described in the Methods.

The secondary structure of atTic20 was also determined after reconstitution into liposomes (Figure 3B). Far-UV CD spectra of reconstituted atTic20 in POPC liposomes showed an enhancement in molar ellipiticity. A slight red shift of the local minimum at approximately 220 nm was also observed in the CD spectrum of atTic20 following reconstitution into POPC vesicles. Comparison of the CD spectra of atTic20 in ZW 3-14 to that of the protein in liposomes reveals that the two prominent local minima of the protein in detergent (~222 nm and ~208 nm) have shifted towards a single minimum (at ~225 nm) and a negative shoulder at ~208 nm in liposomes (Figure 3B). The deconvolution of the CD spectrum of atTic20 in the POPC liposomes revealed a helical content of ~74% (Table 1), which is in good agreement with the helical content predicted from the amino acid sequence (Table 1). In conclusion, atTic20, extracted and purified from bacterial membranes using ZW 3-14 detergent, exhibited a highly helical secondary structure following reconstitution into POPC lipid vesicles.

### Associated state of atTic20 monitored by CD spectroscopy and semi-native SDS-PAGE

**Figure 4:**
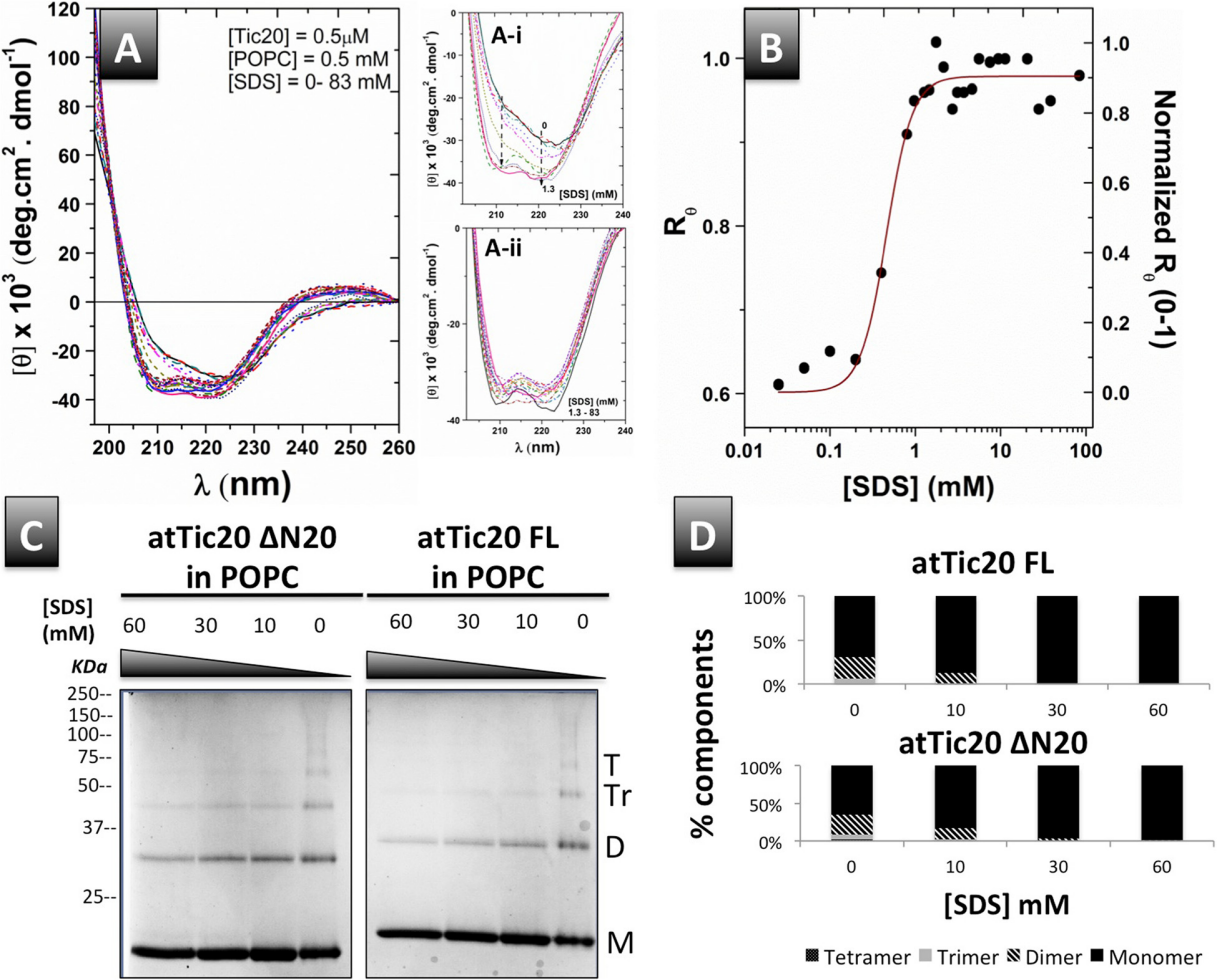
**Titration of atTic20 in POPC liposomes with SDS. (A)** Far-UV CD spectra of reconstituted atTic20 in POPC liposomes titrated with SDS (0 – 84 mM). (A-i) shows the low [SDS] region of the spectra in greater detail (0-1.3 mM), where higher SDS concentrations enhanced negative ellipticities at both 208 and 222 nm. (A-ii) shows the high [SDS] region (1.3-84 mM) in greater detail, where increased SDS concentrations resulted in only small changes in the molar ellipticity of atTic20. **(B)** Plot of R_θ_ *vs*. [SDS] reveals a cooperative dissociation of associated atTic20 in the presence of SDS. The R_θ_ was normalized and K_1/2_ ([SDS] at 50% protein dissociation) was calculated using the Hill fitting (K_1/2_ = 0.5 ± 0.05 mM). **(C)** Semi-native PAGE (12%) of atTic20 and atTic20ΔN20 in POPC liposomes in the presence of increasing concentrations of SDS added to the sample buffer (the electrophoretic running buffer contained 2 mM SDS in all cases). The gels were stained with Coomassie Brilliant Blue. Monomeric (M), dimeric (D), trimeric (Tr) and tetrameric (T) forms of atTic20 and atTic20ΔN20 are indicated. **(D)** Relative proportion of the different associated states of atTic20 and atTic20ΔN20 measured during the SDS titration as determined by measuring band intensity on the semi-native PAGE gels shown in C. Band intensity was quantified using Quantiy One (Bio-Rad) as explained in the Methods.

atTic20 extracted and purified from bacterial membranes using mild detergent or reconstituted into liposomes both displayed high helical contents as reveled by CD spectroscopy (Table 1). In liposomes, atTic20 displayed a marked enhancement in helical content and its CD spectrum displayed the characteristics of α-helices, with the π → π* exciton split bands at ~190 and 208 nm and the n → π* transition at ~222 nm (Figures 3B and 4A). However, compared to a typical α-helical CD spectrum, the parallel band at ~ 208-210 nm exhibited a shoulder-like minimum ellipticity (Figures 3B and 4A). The θ_208_/ θ_222_ ratio of the atTic20 spectrum in POPC vesicles is less than 1 (Figures 3B and 4B). Such low molar ellipiticity ratios between 208 and 222 nm have been used to signify coiled coil motifs or oligomerization of monomers and/or packing of helical domains within a monomer for other proteins [28-31]. We therefore used semi-native PAGE as a method to examine whether atTic20 reconstituted into liposomes displayed characteristics of an oligomeric protein. Semi-native PAGE revealed a mixture of oligomeric forms of atTic20 at the lowest levels of SDS tested (Figure 4C). Monomers and dimers were most prevalent at all concentrations of SDS. The low but detectable amounts of trimers and tetramers were eliminated as the concentration of SDS was increased, such that primarily monomer, and a very small amount of dimer, were visible at the highest concentration tested (Figure 4C and D). Thus, the ability of atTic20 to self-associate in liposomes has been observed in vitro for the first time. It is important to note that reconstituted atTic20ΔN20 also showed evidence of self-assembly, as revealed by CD spectroscopy (data not shown) and semi-native PAGE (Figure 4C).

The dissociation/unfolding pathway and stability of atTic20 in liposomes was further analyzed by using CD spectroscopy to monitor the protein conformation during an SDS titration of atTic20 reconstituted into liposomes. CD spectral analysis revealed two conformational states of the protein when SDS was added (Figure 4A). At the lower concentration range of SDS (0 – 1.3 mM), far-UV CD spectral shape of reconstituted atTic20 displayed a drastic change as SDS was added to the proteoliposome mixture. The negative ellipticities at 210 nm and 222 nm were both enhanced, and the negative shoulder at 210 blue-shifted towards 208 nm and gradually became a visible local minimum (Figure 4A-i). These results suggest a transition of reconstituted atTic20 towards a less constrained conformation with fewer intermolecular interactions between helices and more independence for monomers. Further increasing the SDS concentration above 1.3 mM (to 84 mM) did not produce significant changes in the far-UV CD spectral shape of atTic20; the protein’s CD spectra remained unchanged with two negative maxima at 208 and 222 nm. Plotting the negative ellipticity ratio of these two maxima (R_θ_) as a function of SDS concentration revealed a two-state dissociation of atTic20 in POPC with a dissociation constant K_1/2_ (50% dissociated) of 0.5 ± 0.02 mM SDS (CMC of SDS in buffer was calculated to be ~ 2 mM, data not shown). The CD data are consistent with the semi-native PAGE analysis, which showed that the addition of SDS resulted in simultaneous loss of higher order oligomers (tetramers, trimers, and dimers) from the liposomes and concurrent enrichment of monomers (Figure 4C). The relative amounts of the different oligomeric states of atTic20 were calculated based on protein band intensities on the semi-native PAGE gel (Figure 4C). At the lowest concentration of SDS used (0 mM in the sample buffer, 2 mM in the running buffer), approximately 65% of the protein was in the monomeric form, ~26% was dimeric, ~7% trimeric, and only ~2% was tetrameric (Figure 4D). The tetramers were no longer detectable when 10 mM SDS was included in the sample buffer, and at concentrations of 30 mM SDS and above, both atTic20 and atTic20ΔN20 were almost exclusively found as a monomers, with only trace amounts of dimer detectable (~1%) at 60 mM SDS (Figure 4C and D).

Collectively, the spectroscopic measurements and semi-native SDS-PAGE data suggested that both atTic20 and its N-terminally truncated mutant were able to self-associate in lipid bilayers under our experimental conditions. The oligomers dissociated readily in SDS, suggesting that the interaction between monomers was weak. Additionally, the spectroscopic two-state dissociation of the protein from liposomes during SDS titration, supported by the semi-native PAGE data, implies that the monomeric and dimeric states of the protein predominate. Overall, the combination of experimental techniques used in this study provide evidence that atTic20 has the propensity to form oligomers in a lipid environment.

### The N-terminal domain of atTic20 is intrinsically disordered

**Figure 5:**
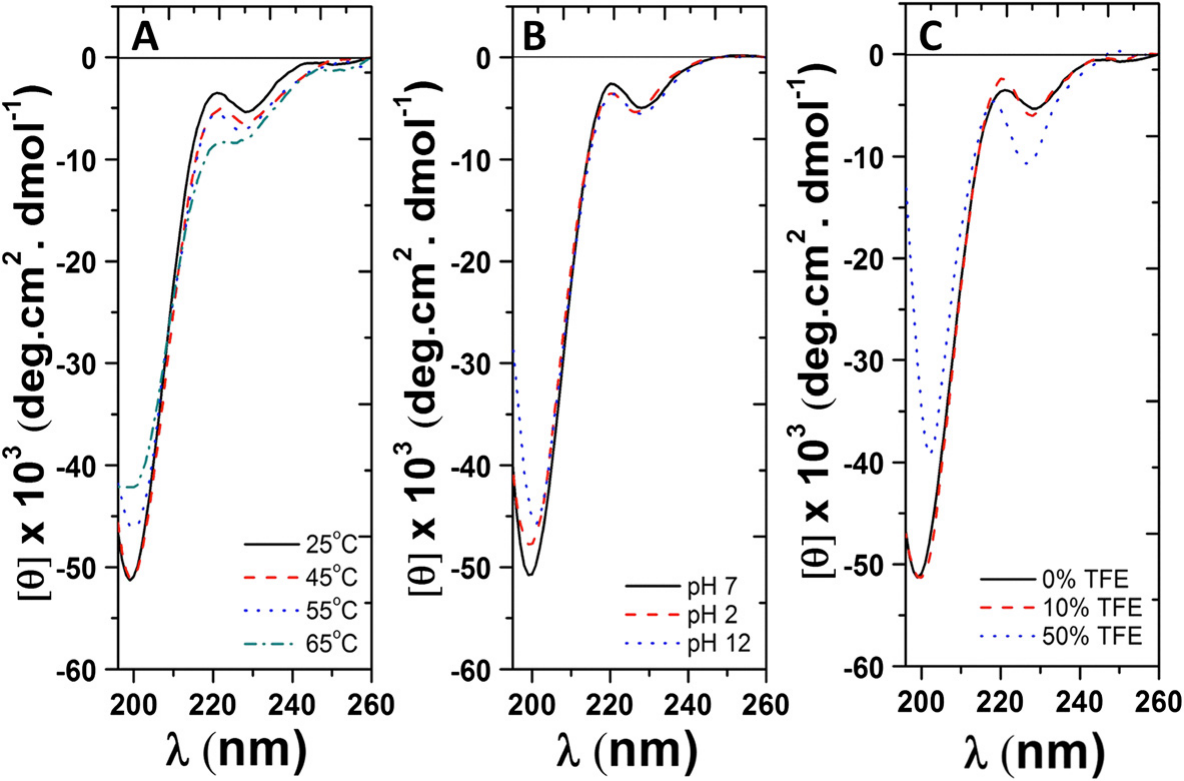
**A synthetic peptide corresponding to the N-terminal domain of atTic20 has characteristics of an intrinsically disordered protein. (A)** Temperature-dependent far-UV CD spectra of N-terminal peptide of atTic20. **(B)** pH-dependent far-UV CD spectra of N-terminal peptide of atTic20. **(C)** Far-UV CD spectra of N-terminal peptide of atTic20 in the absence and presence of 10% and 50% TFE at 25°C. Peptide content was kept at a concentration of 40 μM in Tris buffer for all measurements. The peptide gained modest amounts of structure with increasing temperature, pH, and TFE concentration, which is consistent with the domain being intrinsically disordered.

To examine the secondary structure of the extramembrane N-terminal domain of atTic20, CD spectroscopy of a corresponding synthetic peptide (Figure 1A) was carried out at various temperatures, pHs, and buffer conditions. Under non-denaturing conditions (100 mM NaCl, 20 mM Tris-HCl, pH 7.5, and 25°C), the peptide exhibited a CD spectrum typical of disordered proteins, indicated by the presence of a deep minimum at ~ 200 nm (Figure 5A) [32]. It is noteworthy that the negative ellipticity at ~ 225 nm could be attributed to the close proximity and interaction of Trp residues present at the C-terminus of the peptide [33]. This spectroscopic result supports our computational prediction that the N-terminal domain of atTic20 is disordered under normal physiological conditions (Figure 1Bi).

The structural properties of the N-terminal domain of atTic20 were further characterized through its changes at different temperatures, pHs, and increasing concentrations of trifluoroethanol (TFE) (Figure 5) [32,34,35]. Comparing the far-UV CD spectra of the peptide at different temperatures revealed a modest gain of peptide structure as temperature increased, revealed by an enhancement of the negative ellipticity at ~ 220 - 225 nm (Figure 5A). The influence of Trp-Trp interactions on the peptide's CD spectra might also be lessened at higher temperatures, as indicated by the slight blue shift of the negative ellipticities at ~ 225 nm. In addition, a reduction of the negative minimum at ~ 200 nm revealed a decrease in random coil content of the peptide (Figure 5A). Thus, an increase in temperature induced partial folding of the N-terminal domain of atTic20. In addition, a slight gain in the peptide structure was also observed at high pH (~12) (Figure 5B). Carrying a number of basic amino acid residues, the N-terminal peptide possesses a large positive charge at neutral pH (pI ~10.3). Under basic conditions, this net charge would be minimized, thus reducing the electrostatic repulsion between amino acid residues and inducing partial folding of the peptide [35]. Finally, the secondary structure stabilizer TFE enhanced the N-terminal peptide structure (Figure 5C). At 50% TFE, a notable increase in secondary structure was observed, indicated through the enhancement of local minima at ~ 225 nm and ~ 200 nm. Overall, the N-terminal domain of atTic20 displayed an unstructured conformation with the propensity to gain structure depending on its environment, which is consistent with being characterized as an intrinsically disordered protein domain. The ability to adopt different conformations may play a role in its physiological function(s).

## Discussion

A novel method for expressing and purifying recombinant atTic20 in a native-like folded form is reported in the current study. In addition, the self-association of atTic20 in mild detergent and lipid systems was observed in vitro for the first time, using a combination of biochemical and biophysical techniques, and evidence is presented that the extramembrane N-terminal domain has properties of an intrinsically disordered protein. The evidence for oligomerization may be important when exploring the hypothesized function of the protein as a preprotein channel of the Tic complex.

### Novel recombinant protein expression system for atTic20

Several studies, including ours, predict atTic20 to contain four TM domains that span the chloroplast inner envelope membrane [1,9,10,12,15]. The hydrophobic nature of the protein has made it difficult to produce recombinant Tic20 until recently, when Kovacs-Bogdan et al. reported obtaining atTic20 in bacterial inclusion bodies [15]. A disadvantage of extracting proteins from inclusion bodies is that many such proteins have been shown to be only partially folded [36]. Other disadvantages of this method include the heterogeneity of protein conformations that can be obtained when refolding proteins from inclusions bodies, and the limited number of detergents suitable for the solubilization and subsequent refolding of the protein [37]. In the current study, using a mild auto-induction expression system [24], together with nutrient-rich media, we were able to heterologously express atTic20 in *E. coli* in such a way that some of the protein was directed to the bacterial membranes, even though the majority was still directed to inclusion bodies (Figure 2A and see Additional file 1). Targeting to the bacterial membrane occurred without introduction of a signal sequence onto the N-terminus of atTic20. Although we did not attempt to characterize the nature of the targeting, it is known that the signal recognition particle (SRP) targets multi-spanning transmembrane (TM) domain proteins to the membrane in *E. coli.* SRP is able to recognize either cleavable N-terminal signal sequences or internal TM domains (signal anchor sequences) for targeting proteins to bacterial membranes, and insertion can occur via the SecYEG or YidC translocon [38,39]. Furthermore, SRP is known to be recruited by the presence of tryptophan-enriched transmembrane helices, such as is found in the first predicted TM domain of atTic20 [38]. It therefore seems likely that atTic20 was targeted to the bacterial membrane in an SRP-dependent manner, with the first TM domain serving as a signal anchor sequence. In summary, production of the recombinant protein relied on using the “Codon Plus” strain of *E. coli*, much like Kovacs-Bogdan et al. [15], who used a codon-optimized cDNA for expression of atTic20 in inclusion bodies. Targeting of the protein to the bacterial membrane rather than to inclusion bodies was presumably a result of using the “mild” auto-induction method. In addition, the method resulted in the purification of recombinant atTic20 in detergents in relatively high yield (~1 mg pure protein/L bacterial culture). Both atTic20 and its truncated version (atTic20ΔN20) were shown to retain high helical contents following their extraction from the membrane using 0.1% of the mild detergent ZW3-14 (Table 1). The helical content of the protein (74%) following reconstitution into POPC liposomes was very similar to that predicted from the primary sequence (Table 1). Thus, the expression and reconstitution protocol described in this study is appropriate for preparing highly pure and native-like folded protein that can potentially be used for obtaining high-resolution structures of atTic20. The method might also be applied to other TIC components, whose structural and functional features still remain elusive. In the study reported by Kovacs-Bogdan et al. [15], psTic20 (which shares 60% sequence identity with atTic20) expressed in a cell-free system also exhibited a highly helical secondary structure (78% helix) in the presence of 0.8% Brij-35 detergent (above the CMC of 0.011%). The far-UV CD spectrum of psTic20 reported in that study was indicative of a monomeric conformation, featuring two minima at 210 and 222 nm and a positive maximum at 193 nm [15]. Similar CD spectral properties were also observed in the current study for dissociated atTic20 following the titration of protein from POPC liposomes with SDS (Figure 4A). Therefore, we conclude that expression of atTic20 in bacterial membranes allows for the formation of oligomers that aren’t formed when the protein is expressed in a cell-free environment in the presence of detergent.

### Self-association of atTic20

The recent report that Tic20 comprises part of a 1 MDa complex, together with new components Tic214, Tic100 and Tic56, that forms preprotein-conducting channels independent of Tic110, has contributed to a debate about the stoichiometry of the components in the TIC translocon [5,14,15]. One issue in particular that has been raised is whether Tic20 is present in sufficient quantities to serve as the primary channel forming protein [15]. And if it is the primary channel forming protein, the mechanism by which it operates is still unknown. Determining the structure and oligomeric state of atTic20 represents an important step toward answering these questions.

**Figure 6:**
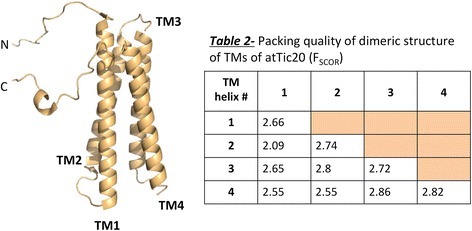
**Possible helix dimerization of atTic20.** A 3-D model of atTic20 was generated using the Distill program [42] as described in the Methods section. Each TM helix shown in the model was used in the analysis of predicted TM helical dimer conformations (PREDDIMER). For each predicted dimer conformation, F_SCOR_ is evaluated, representing the packing quality of helices. A higher F_SCOR_ reflects a high packing efficiency of the dimer [43]. The detailed prediction method is described in the Methods.

In this study, we report the observation that atTic20 has the capacity to self-associate in lipid membranes (Figures 2, 3, and 4). This homo-oligomerization of atTic20 was detected for protein extracted directly from bacterial membranes using mild detergent (Figure 2A), and when reconstituted into liposomes (Figure 4). The predominant associated form of atTic20 was dimeric, although higher-order oligomers (trimer and tetramer) were also observed at much lower levels (Figure 4). Under our experimental conditions, atTic20 oligomeric forms in liposomes could be dissociated with relatively low concentrations of SDS (Figure 4). This relatively weak self-association of atTic20 could be an indication that the oligomeric state of the protein is dynamic, with the ability to associate and dissociate readily in response to particular signals. In the context of the TIC complex at the chloroplast, one such signal could be the presence of preproteins, which has been shown to trigger Tic complex assembly [1]. The potential function(s) of the different oligomers, or which one(s) might be more prevalent in vivo, remains to be determined; however, Kikuchi et al. [5] hypothesize that the 1 MDa complex isolated from chloroplast inner envelope membranes that interacts with translocating preproteins may contain 3 copies of atTic20. It is possible that an oligomer of atTic20 (possibly a trimer) forms the core of the 1 MDa complex and provides the conduit through which preproteins travel. It is also possible that Tic20 operates in a manner similar to Tim23, the mitochondrial translocase component with which it shares sequence and topological similarities, and that is the central component of the TIM23 complex [9,17]. Interestingly, these mitochondrial transporters are dimeric in their functional state [40], which is comparable to the results of this study, underscoring the existence of oligomeric forms of atTic20 in equilibrium with its monomeric form in membranes. Evidence in support of the propensity of atTic20 to form oligomers (specifically dimers) was provided by the transmembrane helical dimer prediction program PREDDIMER (Figure 6) [41]. While no dominant set of pairings emerges from this analysis, the results from the PREDDIMER prediction suggest the possibility of different sets of distinct helix-helix alignments, allowing for multimeric forms of atTic20 to adopt several conformations.

This work also revealed some of the structural features of atTic20 (Figure 1A). The N-terminal peptide preceding the 4 predicted TM domains of the protein displayed characteristics that are consistent with instrinsically disordered proteins, exhibiting flexible conformations in different environments (Figure 5). This conformational elasticity could be important for the physiological function(s) of atTic20, such as peptide recognition or interaction with protein partners in the TIC complex. These protein partners potentially include the other members of the recently discovered 1 MDa complex of the inner envelope membrane (Tic214, Tic100, and Tic56) [5,14]. Interestingly, the truncated version of atTic20, lacking the N-terminal peptide, displayed conformations and showed evidence of self-assembly similar to the full-length protein in detergents and lipid bilayers (Figure 4C). Thus, the ability to self-associate is independent of the N-terminus, and is presumably conferred by one or more of the transmembrane domains. The N-terminal domain may participate in protein interactions with other components of the translocon complex. Of note, is that the extramembrane N-terminal domain of Tim23 has also been reported to be intrinsically disordered; in that case, it is believed that the domain (which is larger than that of atTic20) is involved in preprotein binding [17].

## Conclusion

In conclusion, the novel protein expression system reported in this study resulted in the protein being targeted to bacterial membranes, which is a more biologically relevant system than inclusion bodies, and led to the finding that the protein can exist as a monomer and higher-order associated forms. The existence of associated forms of atTic20 in membranes may be important for understanding its hypothesized function as a preprotein transporter. The observed dimeric, trimeric and tetrameric forms, together with the low dissociation constant (0.5 ± 0.02 mM SDS) of the protein in liposomes implies a relatively weak interaction between the monomers, which in turn suggests a dynamic nature of the equilibrium between the monomeric and associated forms. This may be an indication that changes in the protein's local environment, which could include presence/absence of preproteins, could trigger changes in the multimeric state of the protein. The ability of Tic20 to switch between its monomeric and associated forms is consistent with previous observations that Tic complex assembly is triggered by the presence of preproteins, and that the 1 MDa complex likely contains more than one copy of atTic20, and will be important when testing its hypothesized function as a preprotein channel protein of the inner envelope membrane of chloroplasts.

## Methods

### In silico sequence analysis and structural modeling of atTic20 and its N-terminal peptide domain

Hydrophobicity analysis and transmembrane helix prediction were performed using TopPred server and TMHMM, respectively [25,26]. Three-dimensional prediction of atTic20 structure was achieved using the Distill Server suite and the structure was visualized using Pymol [42,43]. Prediction of intrinsic disorder was completed using IUPred [27]. Helix-helix dimerization and helical motifs were predicted using PREDDIMER [41], TopPred and Distill Server suite. F_SCOR_ was calculated for the TM helical dimer conformations, representing the packing quality of helices. F_SCOR_ is proportional to the relative number of atoms packed within structures, complementarity of hydrophobic properties of helix-helix interfaces, and structural properties of the dimer surface exposed to a lipid environment [41].

### atTic20 and truncated constructs

A cDNA clone encoding pre-atTic20 in pET21a was a gift from Dr. Danny Schnell (University of Massachusetts) [12]. This was modified by PCR to generate a cDNA corresponding to the mature atTic20 protein (amino acid residues 103-274) [12], which was cloned into the XhoI and NdeI restriction sites of the pET21a expression vector (Novagen, Canada), such that it was in-frame with the coding sequence for a C-terminal hexahistidine tag. The construct was introduced into *E. coli* BL21 CodonPlus (DE3)-RIPL (Novagen) for production of the recombinant protein. A cDNA encoding a recombinant version of atTic20 lacking the extramembrane N-terminal amino acids (20 residues) of the mature protein (designated atTic20ΔN20), was similarly cloned by PCR into pET21a. The identity of the DNA constructs was verified by sequencing (The Centre for Applied Genomics, Hospital for Sick Children, Toronto).

### Expression, membrane extraction and purification of atTic20 and its truncated version

Recombinant atTic20 and its N-terminally truncated mutant (atTic20ΔN20) were overexpressed in *E. coli* BL21 CodonPlus (DE3)-RIPL either by the conventional use of 1 mM isopropyl β-D-thiogalactoside (IPTG) at various temperatures (25 and 37°C), or using the auto-induction method [24]. In the auto-induction method, the bacterial culture was grown in the auto-induction media (1% tryptone, 0.5% yeast extract, 1 mM MgSO_4_, 0.5% glycerol, 0.05% glucose, 0.2% lactose, 25 mM (NH_4_)_2_SO_4_, 50 mM KH_2_PO_4_, 50 mM Na_2_HPO_4_) at 22°C for 22 h. Bacterial cells were collected by centrifugation at 5,000×g for 10 min (4°C). The cell pellets were resuspended in extraction buffer (10 mM Tris HCl, 300 mM NaCl) and lysed using a Constant Systems cell disruption press at 20 kPSI in the presence of *cOmplete* protease inhibitor cocktail (EDTA-free) (Roche), DNase (0.5 mg/ml) and lysozyme (0.2 mg/ml). The bacterial cells were fractionated based on the method described by Zoonens and Miroux [44]. Briefly, The cell lysate was cleared of cell debris and inclusion bodies by centrifugation at 20,000×g for 20 min at 4°C. The supernatant was centrifuged at 50,000 rpm (MLA-80 rotor, Beckman-Coulter) for 1 h at 4°C to yield a pellet containing the bacterial membranes. The enrichment of membranes in the pellet fraction was confirmed using an NADH oxidase activity assay (described below).

Bacterial membranes were solubilized in extraction buffer containing 1% (w/v) mild detergents such as LDAO, Triton X-100, Zwittergent 3-14 (ZW 3-14), or CHAPS for 3 h at 4°C prior to purification. The mixture was centrifuged at 10,000×g for 10 min to remove insoluble particulates. The supernatant containing solubilized recombinant protein (atTic20, or its truncated mutant) was then purified using Ni-NTA chromatography under non-denaturing conditions (20 mM Tris-HCl, 500 mM NaCl, 1 mM Tris(hydroxypropyl)phosphine, pH 8.0, 1% detergent). The concentrations of imidazole for binding, washing, and elution steps were 20 mM, 40 mM, and 400 mM, respectively. Purity and quantity of eluted proteins were determined by SDS-PAGE and protein concentration assays (Bradford or Lowry assays), respectively. The pooled eluted fractions containing purified proteins were desalted using Econo-Pac 10DG Columns (Bio-Rad). The final purified protein (~1 mg/L bacterial culture) was stored in desalting buffer (100 mM NaCl, 20 mM Tris-HCl, 1% glycerol, and 1% detergent, pH 8.0) at -80°C.

### Peptide synthesis and purification

A peptide corresponding to the 21-amino acid residues located at the N-terminal of the mature atTic20 (N-ASKDVPSSFRFPPMTKKPQWW-C) was synthesized by standard solid-phase Fmoc chemistry procedures on Wang resin, as previously described [33]. Briefly, HCTU, HOBt, and DIPEA in DMF were used to activate the C-terminus of amino acids, and a 20% piperidine solution in DMF was utilized for Fmoc deprotection. The peptide was purified by a Waters 600E reversed-phase high-performance liquid chromatography (RP-HPLC) system. Peptide identification and purity were examined using analytical RP-HPLC and electrospray mass spectrometry (Waters ZQ4000). A Luna C5 (Phenomenex) column was used for both analytical and semi-preparative RP-HPLC experiments. Concentration of the peptide was determined using Trp absorption at 280 nm.

### Confirmation of protein expression in bacterial membranes

Following the fractionation of *E. coli* to isolate bacterial membranes, Western blot analysis and an NADH oxidase activity assay were used to confirm the presence of overexpressed recombinant atTic20 and the relative membrane enrichment in each bacterial cell fraction, respectively.

For the Western blots, 10 – 50 μg of total protein per fraction were resolved on a 12% SDS-PAGE gel and transferred (110 min, 15 V) to nitrocellulose membranes using the semi-dry technique. The membrane was stained with Amido Black to confirm the efficiency of transfer, and the membrane was blocked overnight at 4°C in TBS-buffer containing 5% skim milk and 0.05% Tween-20. To confirm the presence of hexahistadine tagged recombinant atTic20, or atTic20ΔN20, mouse IgG2b anti-histidine (Millipore) was used as the primary antibody (1:500 dilution). Rabbit peroxidase-conjugated antibody raised against mouse (Rockland) was used as the secondary antibody (1:4000 dilution). Immunodetection was achieved by luminescence using ECL Western Blot reagent (GE Healthcare), and image was captured using Bio-Rad VersaDoc Imaging system.

The relative enrichment of membranes in each bacterial cell fraction was determined using an NADH oxidase activity assay [45]. This membrane-embedded enzyme was analyzed based on its catalytic activity of converting NADH from its reduced to oxidized form, indicative through a decrease in A_340_ of NADH. Briefly, 0.05 mg of total protein from each fraction was added to 12 mM β-NADH in 50 mM Tris-HCl, pH 7.5 and the absorbance at 340 nm was monitored for a period of 15 min (Biotek Syngery microplate spectrophotometer). The rate of decrease in A_340_ is proportional to the NADH oxidase activity [45]. Specific enzyme activity was calculated per mg of protein (ε_NADH_ = 6220 M^−1^cm^−1^).

### SDS- and semi-native polyacrylamide gel electrophoresiss

SDS-PAGE was performed using standard conditions. To provide a “semi-native” condition, the amount of SDS included during PAGE was significantly reduced as compared to traditional SDS-PAGE. The method described by Voulhoux et al. [46] was followed with slight modifications. SDS was included in the electrophoresis running buffer at a concentration of 2 mM (as compared to 35 mM in standard SDS-PAGE). The sample buffer contained 62.5 mM Tris-HCl, pH 6.8, 0.002% bromophenol blue, and 10% glycerol, and concentrations of SDS ranging from 0-60 mM (see figure legends for exact concentrations). Samples were not heated, but were incubated at room temperature for 5-10 min prior to loading the gel. The gels were stained with Coomassie Brilliant Blue R-250, and the relative amounts of protein in each band were determined using Quantity One software (Bio-Rad), after correcting for background intensity of the stained gel. The relative proportion of protein in each band was calculated as a percentage of the total intensity of all bands in each sample.

### Reconstitution of atTic20 and atTic20ΔN20 into liposomes

Following purification, recombinant atTic20 and atTic20ΔN20 were reconstituted into liposomes consisting of POPC (Avanti Polar) for spectroscopic studies, as previously described for other α-helical membrane proteins [28]. Briefly, lipids were dissolved in chloroform, dried overnight under vacuum, and rehydrated in the reconstitution buffer (100 mM NaCl, 20 mM Tris-HCl, pH 8.0). Multilamellar phospholipids were solubilized in C_8_E_4_ (Bachem) to a final detergent/phospholipid ratio of 2.5 by weight. Purified proteins were then added to the mixed lipid/detergent micelles and incubated for 1 h at 4°C. Protein-free liposome controls were prepared in parallel for all experiments. Liposomes or proteoliposomes were formed spontaneously following detergent removal by SM-2 Biobeads (BioRad).

### CD spectroscopic measurements

Far-UV CD spectra of atTic20, its truncated protein and its N-terminal peptide were measured on an Aviv 215 spectropolarimeter (Aviv Biomedical, NJ). Ellipticities are reported as mean residue ellipticity, [θ]. Far-UV CD measurements were carried out in 0.1 cm-pathlength quartz cells, at 1 nm resolution (25°C). The reported spectra are an average of at least eight scans. Secondary structure content of proteins was estimated from backbone CD spectra using the deconvolution program CDSSTR and the analysis was based on a set of 48 reference proteins and performed on the Dichroweb web site [47,48].

## Availability of supporting data

The data supporting the results of this article are included within the article and the Additional file 1.

## Additional file

Additional file 1:
**Protein content of bacterial cell fractions and purified atTic20.** Table summarizing the protein content of bacterial cell fractions and purified recombinant atTic20.

## Abbreviations

CD: Circular dichroism
CHAPS: 3-[(3-cholamidopropyl)dimethylammonio]-1-propanesulfonate
CMC: Critical micelle concentration
C_8_E_4_: Octyltetraoxyethylene
DIPEA: N,N-diisopropylethylamine
DMF: N,N-Dimethylformamide
DTT: Dithiothreitol
HCTU: (2-(6-chloro-1H-benzotriazole-1-yl)-1,1,3,3-tetramethylaminiumhexafluorophosphate)
HOBT: 1-hydroxybenzotriazole
IDP: Intrinsically disordered protein
IPTG: Isopropyl-β-thiogalactopyranoside
LDAO: Lauryldimethylamine-N-Oxide
POPC: 1-palmitoyl-2-oleoyl-*sn*-glycero-3-phosphocholine
SDS: Sodium dodecyl sulfate
SRP: Signal recognition particles
TFE: Trifluoroethanol
TM: Transmembrane domain
TIC: Translocon at the inner envelope membrane of chloroplasts
TOC: Translocon at the outer envelope membrane of chloroplasts
ZW 3-14: Zwittergent 3-14
