# Supplementary material for: Folding and self-association of atTic20 in lipid membranes: implications for understanding protein transport across the inner envelope membrane of chloroplasts
Source: BMC Biochem. 2014 Dec 31;15:29. doi: 10.1186/s12858-014-0029-y (PMC4307631; doi:10.1186/s12858-014-0029-y)
Supplement: Additional file 1: — Protein content of bacterial cell fractions and purified atTic20. Table summarizing the protein content of bacterial cell fractions and purified recombinant atTic20. [file 12858_2014_29_MOESM1_ESM.pdf]

SUPPLEMENTAL INFORMATION

Folding and self-association of atTic20 in lipid membranes: implications for understanding protein transport across the inner envelope membrane of chloroplasts

James Hugh Campbell[1,†], Tuan Hoang[2,3,†], Masoud Jelokhani-Niaraki[2,3], and Matthew D. Smith[1,3*]

Departments of [1]Biology and [2]Chemistry & Biochemistry, Wilfrid Laurier University, Waterloo, ON
[3]Biophysics Interdepartmental Group, University of Guelph, Guelph, ON

†These authors contributed equally to this work

*Correspondence should be addressed to: Wilfrid Laurier University, 75 University Avenue West, Waterloo, ON, N2L 3C5, Canada. Tel: +1-(519)-884-0710 ext 2916, Fax: +1-(519)-746-0677, Email: msmith@wlu.ca

Keywords: Tic20; TIC; Protein self-assembly; Circular dichroism; Protein folding; Structure-function relationship; Protein reconstitution; Chloroplast membrane proteins

---

Table S1. *Total protein content of fractions obtained from E.coli cells expressing recombinant atTic20 **

Fraction	Protein content
Total soluble protein	0.72 ± 0.2 g
Total inclusion body protein	6.8 ± 1 g
Total membrane protein	1.2 ± 0.3 g
atTic20 extracted from membranes using 0.1% ZW 3-14, and purified using Ni-NTA chromatography	1 ± 0.2 mg

*Results are reported per L of culture.
